# Using Goal Achievement Training in juvenile justice settings to improve substance use services for youth on community supervision

**DOI:** 10.1186/s40352-018-0067-4

**Published:** 2018-04-30

**Authors:** Jacqueline Horan Fisher, Jennifer E. Becan, Philip W. Harris, Alexis Nager, Connie Baird-Thomas, Aaron Hogue, John P. Bartkowski, Tisha Wiley, The JJ-TRIALS Cooperative

**Affiliations:** 1 The National Center on Addiction and Substance Abuse, 633 Third Avenue, 19th Floor, New York, NY 10017 USA; 2 Texas Christian University, 3034 Sandage Avenue, Fort Worth, TX 76129 USA; 3 Temple University, 1115 Polett Walk, Philadelphia, PA 19122 USA; 4 Mississippi State University, 1 Research Blvd., Suite 103, Starkville, MS 39759 USA; 5 University of Texas at San Antonio, One UTSA Circle, San Antonio, TX 78249 USA; 6 National Institute on Drug Abuse, 6001 Executive Boulevard, Room 5191, Bethesda, MD 20892 USA

**Keywords:** Adolescent substance use, Juvenile justice, Agency partnerships, Continuous quality improvement, Goal achievement training, Goal achievement training, Implementation science, Behavioral health

## Abstract

**Background:**

The link between substance use and involvement in the juvenile justice system has been well established. Justice-involved youth tend to have higher rates of drug use than their non-offending peers. At the same time, continued use can contribute to an elevated risk of recidivism, which leads to further, and oftentimes more serious, involvement with the juvenile justice system. Because of these high rates of use, the juvenile justice system is well positioned to help identify youth with substance use problems and connect them to treatment. However, research has found that only about 60% of juvenile probation agencies screen all youth for substance involvement, and even fewer provide comprehensive assessment or help youth enroll in substance use treatment.

**Method:**

This paper describes an integrated training curriculum that was developed to help juvenile justice agencies improve their continuum of care for youth probationers with substance use problems. Goal Achievement Training (GAT) provides a platform for continuous quality improvement via two sessions delivered onsite to small groups of staff from juvenile justice and behavioral health agencies. In the first session, participants are taught to identify goals and goal steps for addressing identified areas of unmet need (i.e., screening, assessment, and linkage to treatment services). In the second session, participants learn principles and strategies of data-driven decision-making for achieving these goals. This paper highlights GAT as a model for the effective implementation of cost-efficient training strategies designed to increase self-directed quality improvement activities that can be applied to any performance domain within juvenile justice settings. Efforts to monitor implementation fidelity of GAT within the specific context of the juvenile justice settings are highlighted.

**Discussion:**

Challenges to setting the stage for process improvement generally, as well as specific hurdles within juvenile justice settings are discussed, as are next steps in disseminating findings regarding the fidelity to and effectiveness of GAT in this unique context.

**Trial registration:**

Clinical Trials Registration number – NCT02672150.

## Background

Adolescence marks a period of transition that typically includes an increase in experimentation and risk-taking behavior, including onset of substance use. Persistent alcohol and drug use among adolescents has been associated with a number of related problems, including poor academic achievement, engagement in dangerous behaviors, fatalities, and increased risk of involvement in delinquent acts (Horan Fisher et al. 2017; Tripodi and Bender 2011). Relatedly, the link between substance use and involvement in the juvenile justice (JJ) system has been well established and operates at many different levels. JJ-involved youth tend to have higher rates of drug use than their non-offending peers. Approximately 70% of JJ-involved youth have prior drug involvement (Belenko and Logan 2003), more than three-quarters of these have recently used substances at the time of their arrest (Zhang 2004), and about one-third meet criteria for substance use disorder (Wasserman et al. 2010). These rates are even higher for serious and chronic offenders (Office of Juvenile Justice and Delinquency Prevention [OJJDP], 2004). Increased substance use can contribute to a higher probability of recidivism, which leads to further, and oftentimes more serious, involvement with the JJ system, thus creating a cycle in which youth can become trapped (Bales et al. 2006).

The JJ system is well positioned to help identify youth with substance use problems and connect them to treatment, not only because of the direct access that JJ staff have to these youth with high rates of substance use, but even more compellingly, because the JJ system is dedicated to monitoring and serving youth in ways that aim to reduce recidivism. Thus, intervening in the cycle of repeated JJ system involvement by identifying substance use and linking youth to needed services is congruent with this central mission. Among those JJ agencies that do provide substance use services to juvenile offenders, however, the lack of standardized federal or state mandates for doing so has resulted in wide variation in agency policies and procedures (Chassin 2008; Young et al. 2007; Young et al. 2006) that are often determined to be haphazard, uncoordinated, and largely ineffective (Nissen et al. 2006).

## The continuum of service provision

**Fig. 1:**
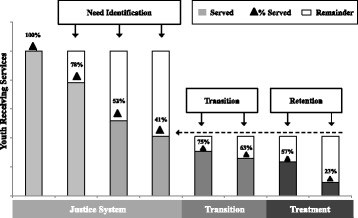
Sample Behavioral Health Services Cascade depicting a site’s rates of screening, assessing, and linking youth to substance use treatment services, as well as rates of treatment initiation, engagement, and completion for youth under community supervision

The continuum of service provision that occurs within and across community agencies has been conceptualized as a Behavioral Health Services Cascade (Belenko et al. 2017). This Cascade captures (1) *Treatment Identification* (i.e., screening and assessing for substance use problems, as well as identifying needed treatment services), (2) *Transition to Treatment* (i.e., referral to a treatment provider and initiation of services), and (3) *Treatment Retention* (i.e., enrolling in and completing treatment). Figure 1 reveals how this data-informed approach to operationalizing service provision can provide a common metric across systems, including JJ, that are responsible for youth health and well-being. Ideally, 100% of youth within an intake cohort [(a) in Fig. 1] would be screened for substance use disorders using evidence-based screening tools. Of youth screened (b), those identified as needing further assessment to determine risk would receive a comprehensive assessment conducted by a licensed clinician (c). Those identified as in need of SU services (d) would be referred to community-based treatment providers (e). The cascade then takes into account the proportion of those youth in need of services who initiate treatment (f), engage in treatment (g), and participate in ongoing continuing care (h). This depiction of the Cascade demonstrates processes that occur internally within JJ agencies (i.e., the proportions of youth who are screened and identified as in need of substance use treatment), those that involve transitioning youth from the care of JJ staff to behavioral health agencies (i.e., the proportions of youth who are referred for and initiate services), and those processes that encompass treatment engagement (i.e., the proportions of youth who enroll in and complete treatment).

In cases of youth placed in residential settings, substance abuse assessment and treatment are likely to have been provided at the facility. Months later, however, these youth return to the community and may need to be linked to a community-based BH provider. At this point, information on a youth’s treatment needs is likely quite rich. The challenge for JJ aftercare agencies, whether probation or parole, is engaging a youth who has been in treatment with a community-based BH provider.

Screening is a quick and economical process for identifying potential behavioral health problems (Wasserman et al. 2003). The availability of paper-and-pencil or computerized measures that can be administered in brief windows of time by laypersons ensures that screening is an efficient means of determining who might benefit from further assessment. A number of evidence-based screening tools are effective in identifying substance use risk among JJ-involved youth (see OJJDP 2004 for specific recommendations). This first step in the service provision process is designed to detect areas of concern that warrant more in-depth analysis by professionals—typically behavioral health clinicians—who have requisite training in identification, prognosis, and treatment planning for a variety of behavioral health problems.

This second step—a comprehensive assessment—differs from screening in that it is conducted by a mental health professional and includes evaluation across a range of problem areas and disorders, general functioning and impairment, and family history, and typically relies on data collected from multiple sources (e.g., the target client and family members; Armstrong and Costello 2002; Grella et al. 2001; Wasserman et al. 2003; Wasserman, Ko, & McReynolds, 2004; Winters et al. 2001). Based on the results of comprehensive assessment, youth who are determined to be in need of behavioral health services are linked to appropriate providers within the community. Despite the availability of evidence-based assessment instruments and treatments, coupled with state or policy mandates to utilize them, most JJ youth with substance use problems do not receive appropriate comprehensive assessment services.

Although current best practice recommendations include the provision of mental health and substance use screening for all youth who come into contact with the JJ system (Chassin 2008; OJJDP 2004), studies have found that only about 60% of agencies screen all youth in their care, and of those who are identified as potentially in need of services, few are provided with a comprehensive assessment or link youth to substance use treatment (Chassin 2008). This problem warrants concern given that substance use disorders are the most common psychiatric problem among justice-involved youth (Teplin et al. 2002) and that the proportion of adolescent offenders entering the juvenile justice system with substance use problems has been consistently increasing over the past few decades (Tripodi and Bender 2011).

To address this gap in service provision to JJ-involved youth, a cooperative research initiative, the Juvenile Justice—Translational Research on Interventions for Adolescents in the Legal System (JJ-TRIALS), was developed among six research centers^1^ and one coordinating center^2^ with the overarching aim of improving the continuum of substance use services for youth under community supervision and, in the process, promoting system-wide change (Knight et al. 2016). To achieve these aims, this study focused on the multilevel nature of service systems and therefore addressed both external (e.g., system-level) and internal (e.g., within service or organization) contexts across stages of organizational change (Aarons et al. 2011).

Here we describe an integrated training curriculum that was developed and implemented as a part of JJ-TRIALS, Goal Achievement Training (GAT), a cost-efficient training in continuous quality improvement strategies that juvenile justice agencies can use to improve their continuum of care for youth probationers with substance use problems. Continuous quality improvement (CQI) has been used to foster improvements in system-level processes in a variety of settings, including welfare, healthcare, emergency response, law enforcement, and education (e.g., Gill et al. 2014; Maxwell et al. 2016; Sun et al. 2016). CQI consists of a broad set of strategies designed to ensure the delivery of services in an efficient and effective manner (e.g., Kerman et al. 2012; O’Neill et al. 2011; Randolph et al. 2012), with the goal of integrating changes to improve both internal processes and external relations.

GAT was delivered onsite as two three-hour training sessions to small groups of staff from juvenile justice and behavioral health agencies. GAT is intended to help JJ agencies determine which areas of the Cascade they wish to improve, teach them evidence-based procedures for improving those areas, and provide tools for evaluating sustained improvement processes and goals after training is complete.

We hasten to note at the outset that the present investigation is a study protocol, not an evaluation. Therefore, in this manuscript, we take special care to delineate the contours of each key component of GAT while providing examples of components as utilized in JJ-TRIALS. We provide sufficient detail so that others who wish to utilize this intervention can replicate—or, as they prefer, adapt—particular facets of GAT as implemented through JJ-TRIALS. We leave evaluation-related matters concerning GAT (e.g., systematic analyses of inputs, outputs, and outcomes) to future manuscripts that are already underway but that are not appropriate for a protocol-based investigation. Consistent with the field of CQI and implementation science, we first establish the parameters of an intervention through protocol publication. Only thereafter do we turn our attention to matters of evaluation. Thus, evaluation questions are being addressed through additional research that should logically follow the publication of a protocol.

## Method

JJ-TRIALS was designed as a cluster randomized trial with a phased rollout, with 34 counties (“sites”) across 7 states randomized into one of two conditions. All sites participated in the *Core* intervention, which consisted of five components offered during the 6-month baseline period of the study: (1) leadership and frontline staff orientation meetings to introduce site stakeholders to the study, (2) an agency-level needs assessment that incorporated process mapping to determine sites’ use of evidence-based screening and assessment for substance use problems and linkage to appropriate behavioral health services for youth at risk, (3) a site-specific report that documented the findings from the needs assessment, (4) ongoing behavioral health training and education for all juvenile justice staff, and (5) the research center-led GAT for a site-selected group of JJ staff representing all levels of employment, along with representatives from the sites’ behavioral health (BH) partners. Half of the sites also received the *Enhanced* intervention, which included continuing support for the use of data-driven decision-making via research staff facilitation over the course of 12 months post-training.

GAT was delivered as a 6-hour onsite training to JJ staff and their BH partners, in which participants learned about effective goal selection and identified a site-specific, shared goal to pursue over the course of the study follow-up period. As part of goal selection support (GSS), sites were encouraged to select a goal that would improve the provision of substance use services (i.e., screening, assessment, and linkage to behavioral health services) to youth under community supervision, based on areas of agency-level deficits as identified by the needs assessment. Following this goal selection, agencies were provided with training in data-driven decision-making (DDDM)—that is, how to review and use agency data to inform decisions made as a part of sites’ efforts to improve Cascade performance (Orwin et al. 2012; Young et al. 2006). The remainder of this paper details the GSS and DDDM curricula and training process, as well as outlines pre-implementation activities, fidelity monitoring practices utilized, implementation challenges, and future plans for evaluating the effectiveness of GAT in the context of JJ-TRIALS. Table 1 depicts the timeline and training agenda for GAT, including pre-implementation and fidelity monitoring activities.

**Table 1:** Timeline and agenda of pre-implementation activities, GAT curriculum, and fidelity monitoring practices

Timeframe	Activity	Participants
Pre-Implementation	Conduct the agency-level needs assessment Draft report based on the needs assessment	Research staff
	Create local change team	JJ & BH staff
	Complete the pre-GAT fidelity assessment	Local change team (LCT)
Implementation	Goal Selection Support (GSS)	Research staff & Local change team (LCT)
	• Review of needs assessment report • Introduce S-M-A-R-T goal criteria • Introduce JJ-TRIALS goal criteria • Select an appropriate goal • Define goal steps	
	Data-Driven Decision-Making (DDDM)	
	• Teach principles of DDDM • Introduce rapid-cycle testing (PDSA) • Share planning worksheets • Review PDSA examples	
Post-Implementation	Complete the GAT fidelity checklists	Research staff
	Complete the post-GAT fidelity assessment	Local change team (LCT)

### Pre-training activities

Success in promoting system change is enriched by a climate supportive of implementation efforts, including identification of key stakeholders who can unite to form a change team to inform the identification of process improvement goals (Becan et al. 2018; Belenko et al. 2013; Hoffman et al. 2012) and participate in an agency-level needs assessment. In JJ-TRIALS, these pre-training activities were designed to be standardized across all sites and are briefly presented here to provide a description of the rich context in which GAT was implemented within the study; additional details associated with these processes can be found elsewhere (Belenko et al. 2017; Knight et al. 2016).

#### The local change team (LCT)

Empirical studies show that collaborations within and across participating agencies, including individuals from relevant disciplines and with varying levels of work experience, can succeed in accomplishing a wide range of process improvement goals (Belenko et al. 2013; Hoffman et al. 2012; Mayer et al. 2011; Shafer et al. 2014; Stummer and Zuchi 2010). Three core features of LCTs are: (1) diverse membership, (2) a focus on data-driven decision-making, and (3) use of external advisors who possess relevant expertise in process improvement (e.g., change team functioning, data use, the evidence-based intervention of interest) (Hagedorn et al. 2006; Saldana and Chamberlain 2012). LCT membership is often intentionally diverse (organizational insiders and outsiders, researchers and practitioners, line staff and supervisors) to foster dialogue and action that balance innovative strategies with practical considerations in the existing organizational culture (e.g., Saldana and Chamberlain 2012; Wandersman et al. 2008).

Within JJ-TRIALS, the LCTs consisted of the individuals within each participating site who attended GAT, selected goals on which to work, and were responsible for carrying out DDDM to develop and, ultimately, roll out system-wide changes to improve their site’s behavioral health cascade. LCT composition in JJ-TRIALS varied somewhat across the participating sites. However, each was primarily composed of 6–8 JJ agency staff with representation from 1 to 2 key community BH agencies. JJ agencies were encouraged to include an executive-level staff member (e.g., county-level administrator with knowledge of resources and policy issues and some power to access resources or shift responsibilities), a quality assurance representative (research staff, data person), supervisors (clinical supervisor, probation officer supervisor), line staff (probation officers, clinical staff when available), and if available, a training representative (to train others within the agency on new procedures).

To promote successful linkages to BH services, JJ agencies were encouraged to identify community-based agencies currently serving as the primary referral sources to the JJ department for youth under community supervision. Including BH partner representation allowed the LCT to target improvement of services specific to youth on community supervision, examine the general pathways in which youth are served between agencies, refine and test actual procedures, and develop a working model to be “scaled out” (Chamberlain et al. 2012) to other BH partners.

#### The agency-level needs assessment and feedback report

Empirical studies indicate that agency-level needs assessments and summary reports with results and recommendations, as informed by a change team and facilitated by an outside party, can be very informative to stakeholders in identifying needs and examining capacity for improvement (e.g., Aarons et al. 2011; Hurlburt et al. 2014). It is often difficult for agencies to assess needs and challenges independently (Lehman et al., 2011). As promoted in other process improvement studies, in JJ-TRIALS, feedback reports incorporated data-centric visuals, such as the Behavioral Health Services Cascade (outlined above; Belenko et al. 2017), and process maps conceptually modeled on visual techniques such as Mapping Organizational Change (MOC; Dansereau and Simpson 2006; Simpson and Dansereau 2007). Process maps have been found to facilitate communication, group focus, and memory across various organizational settings (Newbern and Dansereau 1995). In JJ-TRIALS, process maps visually depict how the JJ system was structured and resourced at the time of the needs assessment and in relation to the Cascade (for more details, see Bowser et al., in preparation). In addition to visually summarizing the most common service route for youth served on community supervision, these reports also captured information regarding (a) the *quality* of currently available services, as determined by state or national accreditation standards and use of evidence-based practices, and (b) contextual features of the site that may influence service implementation (e.g., staffing resources, management information system [MIS] limitations). During GAT, LCT members were encouraged to review the process map and Cascade in order to identify goals for their site to pursue.

### GAT session 1: Goal selection support

The agency-level feedback report provides an example of utilizing data to inform agency decisions, as exemplified through the goal selection support (GSS) curriculum protocol. The 3-hour session has three primary objectives for each LCT: (1) selection of 1–3 goal(s) that can promote movement of youth through the Cascade, (2) identification of steps needed to accomplish each selected goal, and (3) determination of how to measure progress toward each selected goal by creating a written action plan. Sites were encouraged to follow specified criteria in determining their goal(s) and developing their action plan(s). The S-M-A-R-T goal criteria (Lawlor 2012; Locke and Latham 1990; Morrison 2010) were used as a platform for goal selection: Specific: the goal can be broken down into discrete tasks; Measureable: data are available to evaluate change in reaching the goal; Attainable: organization and staff members have ready access to resources needed to address the goal; Relevant: the goal addresses a significant need that can be addressed by the organization; and Time-bound: the goal can be implemented and the change can be evaluated in the time available to the LCT. Each LCT was encouraged to agree upon a finite time period for completion of the goal.

In JJ-TRIALS, each LCT identified at least one process improvement goal to pursue over the course of the 15-month follow-up period. To engender group-based decision-making among LCT members, the GAT facilitator used the Cascade and process maps from the agency-level needs assessment reports to promote a discussion on goal selection using the following common guidelines. Sites were encouraged to select goals that (1) addressed a substantial service deficit along the Cascade as relating to substance use problems among youth at the site, (2) ideally targeted collaboration or transition linkages between the JJ system and the partnering behavioral health treatment providers (whether that provider was internal or external to the JJ system), and (3) met S-M-A-R-T goal criteria (as detailed above).

As part of the GSS curriculum, workgroups were encouraged to use process maps to identify steps/tasks needed to make progress toward site selected Cascade-based goals. Like the overall goal, these tasks or goal steps should meet the S-M-A-R-T criteria. In contrast to the overall goal, then, goal steps are more concrete and manageable in size and scope. Goals steps are intermediate achievements that, when accomplished successively, facilitate overall goal attainment. For example, a site whose goal is to decrease the amount of time between screening all youth for substance-related problems and providing a comprehensive assessment to those identified as high risk might need to first create a shared calendar to use in scheduling assessments with an onsite clinician. For another site whose goal is to increase the proportion of at-risk youth referred by JJ staff to treatment at a partnering BH agency, an intermediate step toward reaching this may include collaborating on the creation of a standardized referral form to be transmitted between JJ probation officers and BH intake staff.

During the GSS session, LCTs were encouraged to articulate all anticipated steps necessary to achieve the selected goal. In addition to identifying the actions involved with each step, LCTs were encouraged to consider parties responsible for the execution of such actions, anticipate due dates and outcomes for all steps, and identify potential challenges that might arise. In JJ-TRIALS, LCTs used a site-specific *implementation action plan* to document and plan these goals steps. Implementation action plans and complementary implementation logs were available to sites following the GAT to monitor incremental progress and completion of steps toward the ultimate site-specified goal. The implementation action plan acted in the form of a contract among all LCT members and outlined procedures for data utilization to track progress toward goals and goal steps. For instance, a site with the goal of increasing youth screening could use the implementation action plan to monitor proportions of youth screened or staff trained on screening or data entry procedures. A sample implementation action plan is depicted in Table 2.

**Table 2:** Sample implementation action plan developed during the GSS portion of GAT

Goal:		By April 2017, the site will double its current referral rate to 80% for youth under community supervision who are in need of substance use treatment services.				
Goal Measure:		The proportion of youth who are referred to treatment.				
Goal Numerator:		The number of youth who are referred to treatment.				
Goal Denominator:		The number of youth who are identified as in need of treatment.				
#	Goal Step	Measure	Numerator	Denominator	Responsible	Due Date
1	Research and purchase an evidence-based screening tool	Complete when screen has been purchased	n/a	n/a	Amy (JJ), Jared (BH)	May 2016
2	Train all JJ staff on how to implement and score the evidence-based screen	Complete when all staff have been trained	# of staff trained	Total # of staff who make referrals	Ted (JJ), Jared (BH)	June 2016
3	Establish and document a formalized intake protocol	Complete when protocol has been drafted	n/a	n/a	Amy (JJ)	Sept 2016
4	Train JJ staff on changes to MIS that incorporate the screen results	Complete when all staff have been trained	# of staff trained	Total # of staff using the MIS	Ted (JJ)	Sept 2016
5	Create a handout of BH options to provide to families during the referral process	Complete when handout has been created	n/a	n/a	Jessica (JJ), Sandra (BH)	Jan 2017
6	Create a standardized notification form to share with BH when making a referral	Complete when form has been created	n/a	n/a		
7	Reconnect with all regional BH partners via in-person meetings	Proportion of BH partners with whom JJ has reconnected	# of local BH partners with whom JJ has reconnected	Total # of local BH partners with whomJJ will work	Cindy (JJ)	Feb 2017
8	Formalize and document communication between JJ and BH partners	Complete when procedures have been documented	n/a	n/a	Amy (JJ)	April 2017

### GAT session 2: Data-driven decision-making

After identifying goals and goal steps, LCTs engaged in a 3-hour session on data-driven decision-making (DDDM). DDDM is a widely used paradigm for system development and improvement (see Berwick 1998; Mandinach 2012; Orwin et al. 2012; Taxman and Rudes 2013; Wisdom et al. 2006). In contrast to top-down decisions imposed on organizations, often motivated solely by management concerns or external guidelines, DDDM refers to the collection, analysis, and interpretation of agency data to objectively inform improvements in policy and practice (Mandinach 2012). Prior research has shown that system or practice improvement is best served by using data to identify problems and monitor progress toward achieving significant goals across a range of settings (e.g., Berwick 1998; Hexom and Menoher 2015; Kaufman et al. 2014).

DDDM is a critical tool in the larger universe of continuous quality improvement (CQI). CQI consists of a broad set of strategies designed to ensure the delivery of services in an efficient and effective manner (e.g., Kerman et al. 2012; O’Neill et al. 2011; Randolph et al. 2012). Therefore, CQI entails the careful monitoring of service processes and outcomes in terms of necessary inputs (e.g., resources, tools, financial investments), outputs (e.g., service number targets, alignment of services with client needs), and outcomes (e.g., program effectiveness, sustained client impact). The overall goal of CQI entails the integration of changes—often implemented in successive iterations—to foster enhancements in internal processes (e.g., case management, coordination among divisions) and external relations (e.g., client satisfaction, interagency collaboration). DDDM has been widely adopted in the corporate world and various segments of the nonprofit sector (e.g., health and welfare organizations, emergency response, law enforcement, and educational institutions) as a means to pursue CQI (e.g., Gill et al. 2014; Maxwell et al. 2016; Sun et al. 2016). DDDM avoids the implementation of changes based on conjecture or anecdotal experiences. With DDDM, empirical evidence is the prime driver of CQI efforts (e.g., Kerman et al. 2012; O’Neill et al. 2011; Randolph et al. 2012; see Kaplan et al. 2010; Solomons and Spross 2011 for reviews). While organizations routinely use data for compliance monitoring and quality assurance at the individual consumer level (e.g., client level services), intentional changes to systems that require aggregate agency-level data to inform process improvement goals are not a routine practice.

**Fig. 2:**
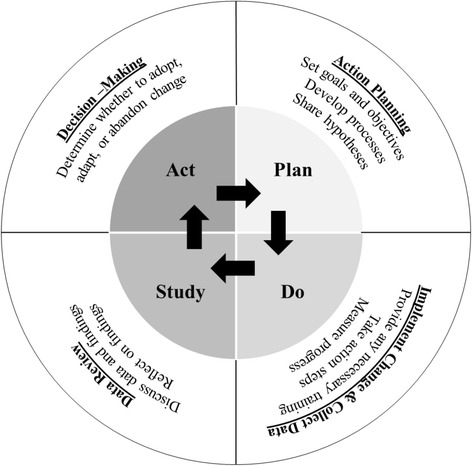
A visual depiction of the heuristic of Plan-Do-Study Act (PDSA) cycles incorporating the use of data-driven decision-making (DDDM)

To assist LCTs in the utilization of DDDM, teams received training on a collection of process maps for conducting *Plan-Do-Study-Act* (PDSA) cycles—an iterative method intended to promote modifications to organizational procedures. PDSA is a common approach to DDDM that promotes a cyclical process of testing out changes on a small subject sample in a narrow time-window, collecting and translating relevant pre-post comparison data into information useful for evaluating effectiveness of the changes, and then scaling out these changes to the organization level (Berwick 1998; Cleary 2015; Wisdom et al. 2006). During the DDDM session, the four stages of the PDSA cycle are reviewed, including processes and decision-making points, use of data at each stage, and how to proceed to the next stage or return to previous stages (see Fig. 2).

#### *Plan* stage

During this first stage of the cycle, the objective is to design a test to investigate a particular question or idea (e.g., change in services). As part of the plan stage, LCT members are to explicitly identify details of the test: (a) what question they hope to answer, (b) who will carry out relevant actions, (c) who will be affected, (d) when and where the test will occur, (e) what data will be collected, and (e) what preparations are needed in advance of carrying out the test. Specification of hypotheses/predicted outcomes and expectations of the test, along with consideration of what data, how it will be collected, and by whom, should be addressed as part of the plan stage.

#### *Do* stage

During the second stage of the cycle, the test is carried out and data are collected. Initial data analysis, along with careful documentation of progress and challenges encountered in the test, should occur during the *Do* stage. This tracking and analysis is useful in informing intermediary decision-making, including whether the test was an obvious success (e.g., there is quick uptake and immediate improvement) or should end early due to insurmountable challenges (e.g., it becomes clear that the test will not work due to lack of resources, buy-in, or comprehension among change agents). Based on these preliminary evaluations, the LCT may decide to end the test early and begin the next PDSA cycle with modifications, roll out the test on a larger scale or under different conditions, or test a different change altogether.

#### *Study* stage

The third stage of the cycle begins after sufficient data are available on a test. During the *Study* stage, the LCT completes analysis of data collected during the *Do* stage and compares results to the hypotheses and predictions made during the *Plan* stage. At this point, relevant observations and challenges are considered. Visual representations of collected data (e.g., charts, graphs) may be useful. The knowledge gained from data analysis and review of challenges and visuals can help determine whether or not the change resulted in substantial improvement, will be useful in the future, and is feasible for scale-up to the system level.

#### *Act* stage

During the final stage of the cycle, the LCT utilizes data collected in the *Do* stage and interpreted in the *Study* stage to make final determinations about whether the tested change should be adopted, adapted, or abandoned. If a test is deemed successful (i.e., resulted in the desired improvement), it may be adopted and implemented on a permanent basis and scaled up to the system level. The decision to adopt a change includes determining the steps to be undertaken to ensure sustainability over time. If a test is deemed as somewhat successful, or if success is anticipated under different circumstances, it may be adapted and retested, based on information learned during the *Study* stage. If a test did not result in meaningful improvement and adjustments are unlikely to predict success in future iterations of testing, then the change may be abandoned. Unless a change is being adapted and retested, a new PDSA cycle based on a different goal or goal step will be started.

For JJ-TRIALS, LCTs received a toolkit with worksheets for each stage of the PDSA cycle and specific examples (as summarized below) highlighting processes and transitions between stages and the iterative nature of the PDSA model as relevant to service changes along the Cascade. The first is an example of a linear progression through the PDSA stages. The second example illustrates the iterative nature of PDSA with a repeated *Study* stage. The third example provides a model for promoting sustainability for changes following PDSA cycle testing.

#### Case example 1: Linear progression through PDSA

In one JJ agency site, the LCT addressed their goal step of increasing the number of youth assessed following a positive screen for a possible substance use problem (*Plan* stage). One probation officer agreed to take on the task of creating a tracking spreadsheet that was designed to help JJ staff better manage the assessment scheduling process. Then another JJ team member used this spreadsheet to track all positive screens and assessments scheduled over a one-month period (*Do* stage). At the end of one month, they found an increase in the number of at-risk youth who received an assessment, but also discovered that probation officers were still not uniformly remembering to schedule a full assessment based on the positive screen results (*Study* stage). For their next cycle, the group decided to focus on developing new procedures designed to remind officers to schedule an assessment whenever there was a positive screen (*Act* stage).

As exemplified above, poor results, often regarded as failures, are invaluable as part of the learning process (Berwick 1998), because they support an understanding of challenges encountered and illuminate possible refinements to both goal steps and changes to promote desired outcomes. A few studies have found that organizations often adopt the PDSA model in theory, but in practice skip the *Study* component, thus undermining the DDDM principles of the PDSA process (Walley and Gowland 2004; Taylor et al. 2013). Therefore, in JJ-TRIALS, the DDDM session placed specific emphasis on using the *Study* stage to promote deliberate, objective, and successful system change.

#### Case example 2: Iterative progression through PDSA

In another JJ agency, the LCT addressed its goal of increasing the proportion of youth referred to treatment by focusing their first PDSA cycle on developing a new referral process whereby probation staff make the initial call to a treatment provider to schedule a family’s initial appointment. To prepare for this new process, one team member created a spreadsheet to track youth who screened positive and were referred for services, and another team member met with probation staff to review the process and answer questions (*Plan* stage). The process was tested over a one-month period, during which staff tracked their progress (*Do* stage); at the end of this test phase, the LCT reviewed the data and determined that, whereas the department was able to increase its rate of referrals as intended, the new process was unexpectedly time consuming, often requiring multiple calls to treatment providers (*Study* stage). To address this issue, the LCT decided to adapt the change by designating one contact person at each treatment provider to call when making referrals in an effort to decrease the amount of time needed to complete each referral (*adapt* decision during the *Act* stage). To prepare for this revised process, a team member consulted with all treatment providers to determine whether a single contact person could be established and modified the spreadsheet to feature this contact information (repetition of the *Plan* stage). Once tested, this change was deemed successful and plans were made to continue this process (*adopt* decision during the repetition of the *Act* stage).

As exemplified here, many PDSA cycles require several iterations of planning, doing, and studying before changes are considered successful enough to adopt during the *Act* stage. For this reason, it is essential that PDSA cycles be conducted on a small subject sample during a narrow time-window before scaling changes up to the organization level (Berwick 1998; Cleary 2015; Wisdom et al. 2006).

#### Case example 3: Sustainment following PDSA

In one JJ agency, the LCT made progress toward its goal of increasing the proportion of youth who receive a comprehensive assessment within seven days of screening positive for substance use problems by adopting a change to their assessment scheduling procedures. The team decided that a positive score on the screen would prompt probation staff to schedule a full assessment, which could be achieved using a shared calendar that indicated available assessment slots, based on the in-house behavioral health provider’s schedule. Scheduling an assessment would prompt probation officers to provide the family with a reminder card that included the provider’s contact information. To ensure that all probation staff were following this new, standardized procedure, the LCT documented the new process in the JJ employee handbook, developed a training plan to train existing and new employees on the new procedures, and devised a measurement system for monitoring the ongoing effectiveness of the new process.

This third case example highlights the transition from the *Act* stage of the PDSA cycle to the *Sustainment* phase of change. The DDDM session of GAT ends with a focus on sustainability by encouraging LCTs to consider how system-level changes can be sustained over time, regardless of staff turnover and revisions to policies and procedures at the federal, state, and county levels. This phase highlights the importance of identifying appropriate standardization, documentation, training, and measurement processes that may include staff training and dissemination of documented protocols and policies, with lessons learned then applied to subsequent change efforts, scale up of changes to other providers or staff, and/or refinement of previously adopted processes across the system.

### GAT Fidelity monitoring

Training fidelity, or the extent to which a training component is administered as intended based on a standardized protocol, is essential for determining training effects (Gearing et al. 2011). Four elements of training fidelity have been identified: design and protocol, training and ongoing supervision, monitoring of delivery, and monitoring of receipt.

#### Design and protocol

The design and protocol of a training are best conceptualized in a training manual that outlines the theory, goals, and strategies, as well as specific details regarding roles and responsibilities, equipment and materials needed, environment, mode of delivery, and troubleshooting techniques (Bellg et al. 2004; Bond et al. 2000; Moncher and Prinz 1991). The design and protocol of GAT in JJ-TRIALS were manualized and provided to all GAT facilitators.

#### Training and ongoing supervision

Fidelity relies on adequate training and ongoing supervision for those responsible for implementing the training, and should take into account individual differences in education and skill levels, experience, and implementation styles (Bellg et al. 2004). Training efforts should be designed to mitigate complexity (i.e., number of components and specificity) and ongoing supervision should include attempts to prevent implementation drift (i.e., deviations from protocols or gradual changes in training curricula; Perepletchikova and Kazdin 2005). In JJ-TRIALS, GAT fidelity procedures included a two-day, in-person tutorial on overall study procedures and on delivery of GAT protocol materials (i.e., GSS and DDDM presentation slides, the agency-level feedback report, the implementation action plan template, and activity worksheets). Ongoing supervision was provided via monthly web-based meetings in which facilitators shared highlights and challenges of implementing GAT across sites.

#### Monitoring of delivery

Training delivery is best monitored by the use of tools that assess the implementation of specific elements of the protocol. Such fidelity measures may include an assessment of relational behaviors or characteristics that are essential for facilitating training success (e.g., warmth, engagement, sensitivity; Gearing et al. 2011). Tools to measure these aspects often incorporate frequency counts or checklists to monitor behaviors and activities, measures of dose (i.e., the amount of content received by participants), and case formulations (i.e., written descriptions of the intervention delivery; Dusenbury et al. 2003). In JJ-TRIALS, training fidelity was monitored via (a) checklists in which trainees and trained observers recorded whether each aspect of the GSS and DDDM protocols was delivered, and (b) monthly web-based meetings conducted with facilitators to query individual experiences conducting the GAT, challenges to intervention delivery, and differences in facilitator-observed training receipt across sites.

#### Monitoring of receipt

Carefully gauging the receipt of training is a component of fidelity that focuses on whether participants comprehend and use the skills imparted during the training session (Bellg et al. 2004; Dusenbury et al. 2003). To assess whether training elements were received, as well as any threats to fidelity in this capacity (e.g., participant resistance, defensiveness, hostility, or impairment), pre- and post-test knowledge measures are ideal (Gearing et al. 2011; Perepletchikova and Kazdin 2005). In JJ-TRIALS, training receipt was monitored via pre- and post-GAT measures as completed by all LCT members. These measures assessed participant knowledge of key learning points from the GSS and DDDM curricula and attitudes toward implementing GAT practices within their respective organizations. As noted more fully below, analyses of the fidelity data collected as part of this project will be the subject of future research.

## Discussion

This paper recounted the ways in which Goal Achievement Training (GAT) was developed and implemented in the context of JJ-TRIALS, a multi-site randomized controlled trial designed to improve service provision in JJ agencies along the Behavioral Health Services Cascade and service linkages with local BH providers. GAT helped JJ-TRIALS sites select process improvement goals and learn how DDDM could be used to pursue those goals, thereby enhancing local systems. The diverse locales in which GAT was implemented provide a stringent test of overall effectiveness as a CQI conduit and its utility in variegated contexts.

The principles promoted within the JJ-TRIALS GAT are designed to encourage members of the LCTs to change how they think about system improvement: (1) from use of data solely for quality *assurance* to an expanded use for quality *improvement*, (2) from dependency on external evaluators for leading change efforts to user-friendly brief, practitioner-led tests of innovations, (3) from mostly top-down decision-making to engaging staff at multiple levels in system improvement, and (4) from perceiving JJ and BH stakeholders as residing in separate spheres of influence to seeing them as collaborators in creating a single system that serves common goals. Although JJ leadership buy-in and willingness to participate in JJ-TRIALS are invaluable in setting the stage for using GSS and DDDM in such process improvement efforts, it is likely that direct training experiences, such as those provided in the JJ-TRIALS GAT, in which process improvement experts provide intensive examples and guidance, play a significant role in system-wide adoption of CQI practices.

### Challenges to promoting system change

Implementation of workgroup activities to promote system change, as part of the JJ-TRIALS project, highlighted a number of challenges—some previously identified within the literature and some unique to juvenile justice settings, operating under specific federal, state, and county legislation. As previously documented in the CQI literature, sites participating in research often struggle with continuing change efforts beyond the period of project involvement (Aarons et al. 2011; Scheirer and Dearing 2011). JJ-TRIALS addressed this critical sustainment problem by developing, training, and circulating tools and structured decision-making processes, offsetting this often encountered resource hurdle. This toolkit contained GSS and DDDM manuals, planning worksheets, and detailed instructions, specific case examples of goal selection and rapid-cycle testing, PowerPoint slides to use in future in-house training, and data tools for tracking progress, such as the Implementation Action Plan for GSS and an Excel dashboard to create visual charts for communicating PDSA results. Nevertheless, even with these tools, a given site may encounter constraints that challenge its ability to sustain CQI endeavors.

Five implementation challenges were identified within JJ-TRIALS sites that are particularly salient to both correctional and behavioral health service contexts. First, some LCTs experienced membership turnover, thus creating shifts in membership or the introduction of members who had missed the initial 6-hour GAT. In response to this issue, a brief self-administered video-based training package was created. The effectiveness and comparability of this modality of GAT has yet to be examined in relation to the in-person, group-based GAT.

Second, some sites struggled with limitations in the ability to document services, access records, and produce reports from their existing case management systems. This problem is not isolated to the JJ system. In particular, data limitations and a lack of data literacy among line staff have been noted previously by DDDM researchers (Mandinach, 2012; Ikemoto and Marsh 2007) and these, along with the absence of staff with analytic capabilities, were noted in several sites, particularly those located in more rural areas. Moreover, the DDDM-based literature emphasizes a number of relevant constraints related to organizational culture, such as varied support from top management (Walley & Gowland, 2004; Chinman et al. 2012; Ikemoto and Marsh 2007), limited time for line staff to participate in CQI efforts (Chinman et al. 2012; Mandinach, 2012; Ikemoto and Marsh 2007), and a lack of experience in collaborating with external agencies (Ikemoto and Marsh 2007). Data limitations, as exemplified in varying settings, including the JJ context, present extreme barriers to fully utilizing DDDM principles.

Third, some sites are constrained in the ability to share information between JJ and BH agencies. While case oversight is provided by the juvenile probation department for youth under community supervision, both the probation department and the BH agency may be providing services and communicating with parents of the youth. It is not uncommon for federal and state policies to limit the types of information that BH agencies can provide to JJ agencies (Fletcher et al. 2009; Gil-Garcia et al. 2005). This constraint can undermine the practice of DDDM when monitoring process improvement goals involves tracking youth transitions between JJ-BH and service provision by BH.

Fourth, and related to broader structural and institutional forces, JJ-BH partnerships can be situated along a rather expansive spectrum. Specifically, some larger JJ systems may employ a BH specialist “in house.” While this arrangement helps create a persistent BH presence in the JJ agency, the magnitude of the BH workload would suggest that the JJ agency could benefit from expanding their linkages to include local BH providers. This structural diversity in JJ-BH partnerships has possible implications for the way trainings to promote process improvement are conducted. For instance, trainings with agencies that rely on “in house” BH specialists may focus more on making changes to internal processes, while trainings with agencies that must rely on outside BH specialists may focus more on developing memoranda of understanding between agencies and on ensuring that data-sharing confidentiality is established and maintained.

Fifth, to promote a manageable group size, JJ agencies participating in JJ-TRIALS were encouraged to restrict BH representation in their workgroup to 1–2 key BH agencies. This recommendation may have prevented LCTs from including the full range of service providers available to JJ agencies within their county and may limit the standardization of practices and policies across BH agencies regarding referral, tracking, and information sharing. Thus, part of the learning process for JJ agencies involved figuring out how to make system-level decisions that were sensitive to the variety of BH agencies in their counties that provide services to JJ-involved youth. Some agencies grappled with the question of whether to invite more behavioral health members to the LCTs and run the risk of letting the LCTs become too large or to keep the team small and therefore potentially be limited in scope.

## Conclusions

The five challenges described above are likely to each manifest in different ways when GAT is delivered in various agencies and municipalities with unique contextual and procedural factors giving rise to a distinctive profile of implementation barriers. The feature of GAT delivery that makes solutions to these unique barrier profiles possible is the flexibility of implementation that can take into account agency and context-specific principles. To facilitate this breadth of implementation, the GAT manual offers concrete examples of methods of problem-solving in the face of specific challenges in order to increase generalizability of the protocol.

This study is characterized, quite intentionally, by one limitation. The focus of our investigation has been an in-depth description of the key components of JJ-TRIALS GAT implementation. We have intentionally left GAT-related implications—including lessons learned about best practices, suboptimal strategies, and implementation beyond juvenile justice agencies—for follow-up studies that reflect squarely on the most significant implications from this implementation. In that future work, we plan to address, among other issues, questions about the optimal uptake of the intervention and prospects for its generalizability. At this juncture, we can say with some confidence that GAT generalizability seems quite promising, based on preliminary work that has been conducted to adapt the protocol for use in diversion programs interested in increasing HIV education and screening.

Although GAT was fielded in juvenile justice agencies, this program was designed quite broadly with human service organizations of any sort in mind. Goal-oriented action and data-driven decision-making have become standard expectations throughout the human service field. GAT could therefore benefit agencies well outside the orbit of juvenile justice (primary care, health promotion, welfare, law enforcement, etc.). To be sure, different examples related to the particular field of implementation would need to be used to resonate most fully with the field in question (e.g., selecting patient outcome goals in primary care medicine). However, we have every reason to believe that the basic architecture of GAT could remain intact if it is transposed to other fields to foster more optimal functioning.

The substantial efforts undertaken by the JJ-TRIALS study design team to monitor implementation fidelity, as outlined above, have resulted in a trove of data that can be used to assess the effectiveness of the GAT design and protocol, training, delivery, and receipt. Future research examining these data will provide valuable insight into stakeholder attitudes and knowledge of goal selection techniques and rapid-cycle testing before and after participation in GAT, LCT cohesion and motivation, sites’ ability to make progress on goal steps and achieve their site-specified goals, and whether sites were ultimately able to effectively increase substance use service provision to youth under community supervision. Findings will aim to contribute to the scholarly literature dedicated to identifying strategies for promoting system-level changes that improve the health and well-being of youth within the unique context of the JJ system.

## Abbreviations

BH: Behavioral health
CQI: Continuous quality improvement
DDDM: Data-driven decision-making
GAT: Goal Achievement Training
GSS: Goal selection support
JJ: Juvenile justice
JJ-TRIALS: Juvenile Justice—Translational Research on Interventions for Adolescents in the Legal System
LCT: Local change team
MIS: Management information system
MOC: Mapping Organizational Change
OJJDP: Office of Juvenile Justice and Delinquency Prevention
PDSA: Plan-Do-Study-Act

## Footnotes

1 Columbia University, Emory University, Mississippi State University, Temple University, Texas Christian University, and University of Kentucky.

2 Chestnut Health Systems
